# Concept of Flocks Fragmentation and Averaging Method for the Application of Electrocoagulation in Process for Coke Oven Wastewater Treatment

**DOI:** 10.3390/ma14216307

**Published:** 2021-10-22

**Authors:** Dariusz Mierzwiński, Przemysław Nosal, Andrzej Szczepanik, Michał Łach, Martin Duarte Guigou, Marek Hebda, Kinga Korniejenko

**Affiliations:** 1 Faculty of Material Engineering and Physics, Cracow University of Technology, Jana Pawła II 37, 31-864 Cracow, Poland; szczepanik@cebea.com.pl (A.S.); michal.lach@pk.edu.pl (M.Ł.); mhebda@pk.edu.pl (M.H.); 2 Faculty of Mechanical Engineering and Robotics, AGH University of Science and Technology, Mickiewicza 30, 30-059 Cracow, Poland; pnosal@agh.edu.pl; 3 Facultad de Ingeniería y Tecnologías, Universidad Católica del Uruguay, B de Octubre 2738, Montevideo CP 11600, Uruguay; martin.duarte@ucu.edu.uy

**Keywords:** electrocoagulation, coke oven wastewater, wastewater treatment

## Abstract

The main objective of the article is to develop the concept of flock fragmentation and the averaging method for the application of electrocoagulation in the process of treating wastewater from coke ovens. The designed solution was part of an innovative system for the coke oven wastewater treatment process. The system is dedicated to removing the hazardous elements and compounds from wastewater from leaching ashes in municipal waste incineration plants. The design of the process and its automatization was based on a quantitative simulation method. The balance equations of mass, energy, and momentum of transport, complemented by the kinetics of the related reaction, are used during the calculation of the process. The main result achieved is a practical solution—the reactor’s scheme, classified due to a patent procedure in the Polish Patent Office.

## 1. Introduction

Coke production requires a huge amount of water for the production process, and large amounts of contaminated wastewater are then discharged into the environment. [1,2]. Coke wastewater is one of the most contaminated and toxic aqueous streams of waste and contains organic as well as inorganic pollutants [3,4,5]. Furthermore, they occur in large amounts, between 0.3 and 4 m^3^ per ton of coke [2,6]. Today, the most important aspect is the development of new and more effective water treatment methods for this industry [6,7,8].

Nowadays, wastewater treatment in coke ovens is based mainly on mechanical-chemical and biological methods [9,10]. The main aim of these processes is dephenolation of wastewater; however, other hazardous organic and inorganic wastes are also removed during this process, such as oils and tars, slurries, free and fixed ammonia, volatile and nonvolatile phenols, thiosulfates, hydrogen sulfide, and highly toxic substances such as cyanides and rhodonites [9,10]. It should be noted that the application of conventional treatment methods, based on activated sludge technology, is not always efficient [1,11]. Because of that, the development of new methods is required. Other motivators for research and development work in this area are cost reduction and a lower amount of waste discharge. Additionally, the existing methods are joined to obtain higher efficiency of the whole process [2,12,13]. Electrocoagulation could be one of the processes applied during wastewater purification and is considered an alternative to the conventional coagulation process. Electrocoagulation has higher efficiency in the removal of target contaminants, including free cyanides, sulfurides, and, especially, complex cyanides than traditional coagulation conducted by chemical processes [1,9]. The advantages also include that it does not cause an increase in the wastewater salinity and it does not require the adjustment of wastewater parameters for efficient performance [9,14]. The electrocoagulation process could be an alternative to chemical coagulation of both water and wastewater [15,16,17], and for selected elements, including heavy metals, it could be the most effective method [18]. Despite the high particle removal efficiency, other advantages associated with the electrocoagulation process include the lack of requiring additional chemicals, the reduction in the volume of sludge produced, and the possibility of complete automation [19,20,21].

Electrocoagulation is a treatment process that uses electrical current to treat and flocculate contaminants without having to add coagulations [16,18]. The first step in this process is the formation of coagulants by electrolytic oxidation of the electrode. Then, destabilization of the contaminants and particulate suspension as well as the breaking of the emulsions take place, and finally, the aggregations of the destabilized particles are created in order to form flocs [18,22,23]. During electrocoagulation, iron or aluminum anodes that are subjected to electrolytic solubility are the most commonly used. In that way, select ions of the mentioned metals are added to water or cleared wastewater, acting as an electrolytic coagulant—an electrocoagulant [23,24,25,26]. As a result of the electrochemical addition of coagulating Fe^2+^ ions, which spontaneously oxidates and creates Fe^3+^, the pH of the system always increases due to cathode oxygen polarization. On the other hand, ‘traditional’ chemical coagulation (hydrolysis of Fe^3+^ (PIX) cations) always decreases pH. In both chemically coagulated and electrocoagulated wastewater, Fe^3+^ cations create positive micelles {Fe(OH)_3_}, which still act as colloidal sorbents [26,27,28].

Currently, the electrocoagulation process is widely used for different types of pollution [29,30,31]. It has found application in many different industries, including the cleaning and neutralization of oil emulsions used in machining and abrasive handling, wastewater from the textile industry, wastewater from agro-food processing businesses, wastewater from car washes, heavy metals, and other pollutants such as coke oven wastewater [32,33,34,35]. Electrocoagulation is a technology based on the interaction between many electrochemistry, coagulation, and flotation processes [36,37]. Thermodynamic modeling of solution chemistry concerning hydrolyzed metal cations, which connects subjects of electrochemistry and coagulation, is a complex engineering task [38,39,40]. From the perspective of describing electrocoagulation as a whole, the problem of mixing hydrodynamics and the kinetics of that process is important. There are many complex physicochemical processes that move a dominant pollution separation mechanism to the area between sedimentation (controlled by gravity) and flotation (controlled by hydrostatic lift) [41,42]. The design of processes and their automatization is based on the quantitative simulation method. The balance equations of mass, energy, and momentum of transport, complemented by the kinetics of the related reaction, are used during the calculation of the process [43,44].

The main aim of the provided research was to develop the concept of flock fragmentation and the averaging method for the application of electrocoagulation in the process of treating wastewater from coke ovens. The article presents the new concept of flock fragmentation and an averaging method for the application of electrocoagulation in the process for the treatment of coke oven wastewater. The innovative aspects of this research are connected to the development of original systems for application in the coke industry as the most effective replacement for traditional technologies. The designed solution was part of an innovative system for the wastewater treatment process in a coke oven. 

## 2. Methods

### 2.1. Granulometry Measurements

The basic input data for the design process was the particle size distribution of the sludge floccules. A device dedicated to particle size distribution analysis—Analysette 2 made by Fritsch (Fritsch GmbH, Idar-Oberstein, Germany)—was used to analyze particle size after an electrocoagulation process. It was applied because the first trials showed the unsuitability of the conventional devices for particle size distribution (PSD) of the sludge floccules due to their high instability and modification of the structure during sample preparation. The wastewater was selected and analyzed after the process. The electrocoagulation process was conducted in a mixing and non-mixing forced flow of liquid. Research shows influences of both the time and the current of the electrocoagulation process on particle creation. The volume of spatial shapes of sediment flocks, which will be used to develop the concept of residuals fragmentation and to average the Sauter diameter of particles in the unit volume of flowing stream, was defined analytically.

### 2.2. Computational Methods Used for Computational Fluid Dynamics Tests and Simulations

The modelling of the stirred tank reactor was conducted in steps. First, the modelling was carried out using the numerical Computational Fluid Dynamics (CFD) analysis of fluid flow during the electrocoagulation process. The purpose of this step was to estimate the physical behavior of fluid flow using numerical methods. The Rocky program was applied to simulate the aggregation of molecules carried by hydroxides onto the liquid surface. Next, the geometry of the mixer, fluid capacity, and baffles in the form of electrodes were modelled as separate regions using Autodesk Inventor software. Next, these geometries were imported into the ANSYS Fluent environment (ANSYS Inc., Canonsburg, PA, USA) to perform preprocessing and discretization. Complex interfaces between contact fluid fields and boundary conditions were made directly in the ANSYS software geometric module. The mesh was generated in order to discretize the computational domain for small control capacities, where basic fluid mechanics equations were approximated with numerical computing. 

## 3. Results and Discussion

### 3.1. The Results of Granulometry Measurements

**Figure 1:**
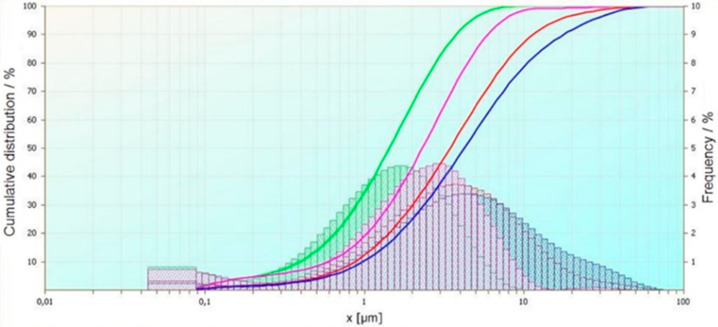
Effect of mixing and oxygenation of selected wastewater during the electrocoagulation process on the size of coagulants.

**Figure 2:**
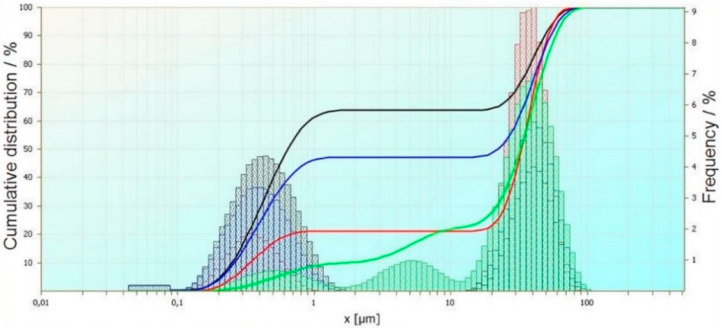
Effect of mixing, oxygenation, and voltage change used in the electrocoagulation process of selected wastewater on the size of coagulants.

**Figure 3:**
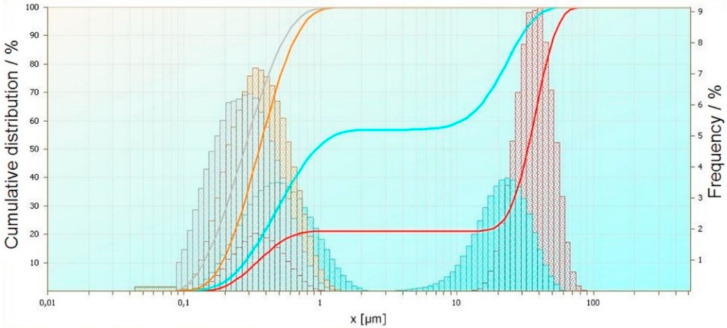
Effect of mixing, pre-aeration, and time change used in the electrocoagulation process of selected wastewater on the size of coagulants.

The measurement of wastewater after an electrocoagulation process has been performed. The experiment was carried out with using potentiostatic modes. Three things have occurred in the process: Time, current density, and oxygenation of the wastewater. The selected wastewaters have been chosen on the basis of checking the number of sulphides and cyanides, as well as their reduction after the process. Based on the current particle size analysis, the results were brought together to show the influence of the process’ time, voltage, and mixing and oxygenation on particle size. The obtained results have been compared and summarized in order to show the influence of selected process parameters on particle size. An exemplary comparative analysis of the results is shown in Figure 1, Figure 2 and Figure 3.

The first summary (Figure 1) shows the influence of the mixing and aeration of wastewater during the process on the properties of the flocks. The green curve is the result of the analysis of wastewater after electrocoagulation. The process parameters were 5 V voltage and a process times of 15 min. The sample was mixed under electrocoagulation. The violet curve shows the results of the particle size analysis after electrocoagulation of the same wastewater but oxygenated. The blue curve is the result of the analysis of wastewater after electrocoagulation. The first step was to aerate the wastewater before electrocoagulation. Parameters of the process were 2 V voltage and a processing time of 15 min. The sample was mixed under electrocoagulation. The red curve shows the results of the particle size analysis after electrocoagulation of the same wastewater but oxygenated.

The second summary (Figure 2) shows the influence of mixing, aeration, and current density on the size of the electrocoagulation flocks. The black curve shows the particle size distribution for 10 V voltage, the blue curve shows the particle size distribution for 7 V voltage, the red curve shows the particle size distribution for 5 V voltage, and the green curve shows the particle size distribution for 2 V voltage. Reducing the voltage at a constant rate causes a reduction in the proportion of particles in the range of 0.1 ÷ 1 μm. The share of particles in the range of 10 ÷ 100 μm grows. The largest share of the largest particles was observed at 5 V voltage. At 2 V, particles sized between 1 ÷ 10 μm were formed, which were not observed in the other cases.

The next summary (Figure 3) shows the influence of mixing, preliminary wastewater aeration, and change in processing time on electrocoagulation flock properties. The grey curve shows the particle size distribution for 5 V voltage and a processing time of 60 min, the orange curve shows the particle size distribution for 5 V voltage and a processing time of 45 min, the light blue curve shows the particle size distribution for 5 V voltage and a processing time of 30 min, and the red curve shows the particle size distribution for 5 V voltage and a processing time of 15 min. Extending the electrocoagulation time at a constant voltage of 5 V causes a reduction in the share of coagulants. The proportion of particles in the range of 10 ÷ 100 µm decreases and the proportion of particles in the range of 0.1 ÷ 1 µm increases. After 45 min electrocoagulation, no particles larger than 1 μm were observed.

### 3.2. Parameters of the Electrocoagulation Process

We assume that in the easiest way possible, a new reactor can be described as a tubular reactor. According to the flow of the plug in a perfect tubular reactor (empty), the real time of the reagent particles equals the mean value of the time Equation (1).

(1) $$\tau =\tau_{z}^{\prime }=\bar{\tau },\rho_{m}=\mathrm{const}$$

It follows that in the reactor mouth, particles with an assumed concentration will appear after the time equal to the reactor mean value. Mathematically, it can be described as the following function Equation (2).

(2) $$F\left(\tau \right)=\frac{C_{A}}{C_{0A}}=\{\begin{array}{c} 0,\;\mathrm{if}\;\tau <\;\bar{\tau }\\ 1,\;\mathrm{if}\;\tau \geq \;\bar{\tau } \end{array}$$

We assume that, in accordance with the theory of impulsive liquid delivery under conditions of plug flow, all of the important particles move without mixing longwise in the reactor and leave after the time described in function in Equation (2), which after modification can be described as function in Equation (3).

(3) $$E\left(\tau \right)=\frac{C_{A}\cdot {\dot{F}}_{0\upsilon }}{n_{0A}}=\{\begin{array}{c} 0,\;\;\mathrm{if}\;\tau \neq \;\bar{\tau }\\ \infty ,\;\;\mathrm{if}\;\tau =\;\bar{\tau } \end{array}$$

According to research and changes in particle size calculated with Analysette 2 made by Fritsch and described in Section 2.1, it was found that the range for analysis should be limited to 30 min. At the current level of research aiming to establish all parameters of the new reactor, the initial model of electrocoagulation kinetics was formulated. They are presented in the following results.

### 3.3. Formulation of the Initial Kinetic Model of the Electrocoagulation Process

To investigate the economy of the process that is developed, the electrical energy consumption was modelled using the equation proposed in Equation (4)

(4) $$E=\frac{U\cdot I\cdot t}{V}$$

where U is the applied voltage [V], I is the current intensity [A], t is the time of the process [h], and V is the volume of the solution [L].

Current work investigates the dependence of electrical parameters (Table 1) on the electrocoagulation process. In addition, it focuses on the first attempt to model the removal of sulfur and cyanides from wastewater.

**Figure 4:**
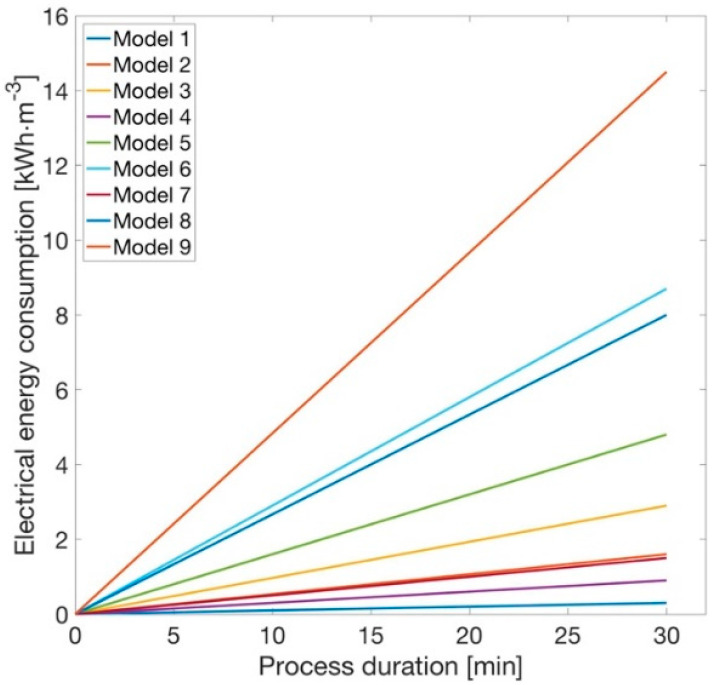
Electrical energy consumption according to the time of the electrocoagulation process.

In this case, using a simplified assumption involving constant values of electrical parameters, it can be observed that the energy consumption increases proportionally to the progressing time of this process (Figure 4).

In the electrocoagulation process, iron electrodes are used, which is why it is appropriate to calculate the loss of its mass. In addition, this variable can be used to determine the rate coefficient in the sulphide concentration function. The amount of mass loss was calculated using Equation (5)

(5) $$M_{\mathrm{Fe}}=\frac{I\cdot t\cdot M}{z\cdot F\cdot V}$$

**Figure 5:**
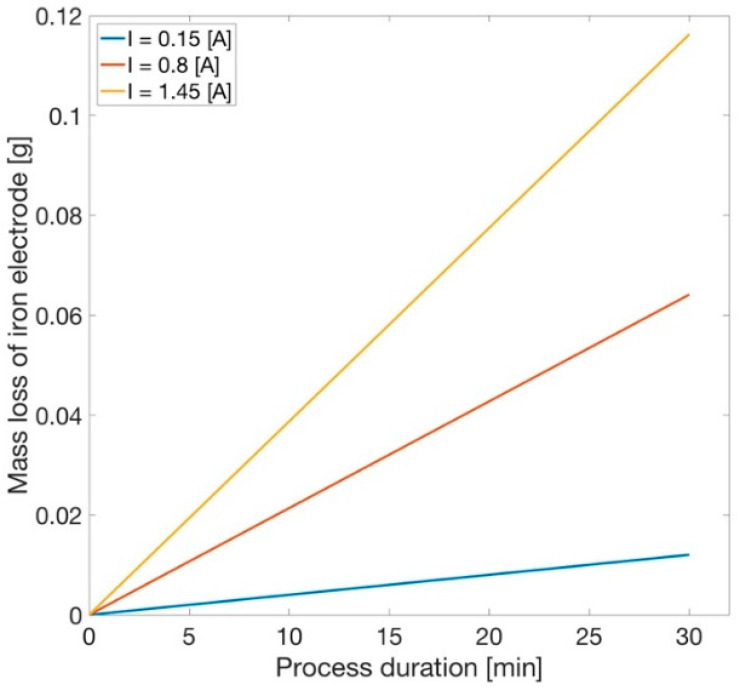
Decrease in the iron electrode mass during the process.

In this equation, the processing time t is given in seconds, M is the molecular mass of iron (g/mol), z is the number of changeable electrons, and F is the Faraday constant (96,485 Cb/mol). The results for the entire process are shown in Figure 5.

The reaction rate with iron ions can be described according to Equation (6)

(6) $$-\frac{{\mathrm{dc}}_{S}}{\mathrm{dt}}=k_{S}\cdot c_{\mathrm{Fe}}\cdot c_{S}$$

where c_S_ is the sulphides concentration, k_S_ is the rate coefficient value, and c_Fe_ is the iron concentration. The equation that describes the removal of sulphides can be simplified to the form Equation (7)

(7) $$-\frac{{\mathrm{dc}}_{S}}{\mathrm{dt}}≅{k^{\prime }}_{S}\cdot c_{S}$$

where the expression k′_S_ was used, which is a complex function for the process velocity. After integration we obtain Equation (8)

(8) $$\ln\left|c_{S}\right|+C_{1}=-{k^{\prime }}_{S}\cdot t+C_{2}$$

and to dispose the logarithm from the formula, the exponential function must be used in Equation (9)

(9) $$c_{S}=C\cdot \exp\left(-{k^{\prime }}_{S}\cdot t\right)$$

Then, to determine the value of the constant, information on the initial conditions was used and inserted into Equation (10)

(10) $$\{\begin{array}{c} t=0\\ c_{S}\left(t\right)=1.74 \end{array}\to C=1.74$$

When the value of the iron electrode mass loss rate AFe was known, then the iron concentration in the tank volume V was calculated in Equation (11)

(11) $$c_{\mathrm{Fe}}=A_{\mathrm{Fe}}\cdot V^{-1}$$

The interim rate coefficient value k′_S_ is computed based on the proposed Equation (12)

(12) $${k^{\prime }}_{S}=X_{S1}\cdot X_{S2}\cdot X_{S3}\cdot c_{\mathrm{Fe}}$$

where:

(13) $$\begin{array}{c} X_{S1}=1\\ X_{S2}=1\\ X_{S3}=U\cdot I \end{array}$$

**Figure 6:**
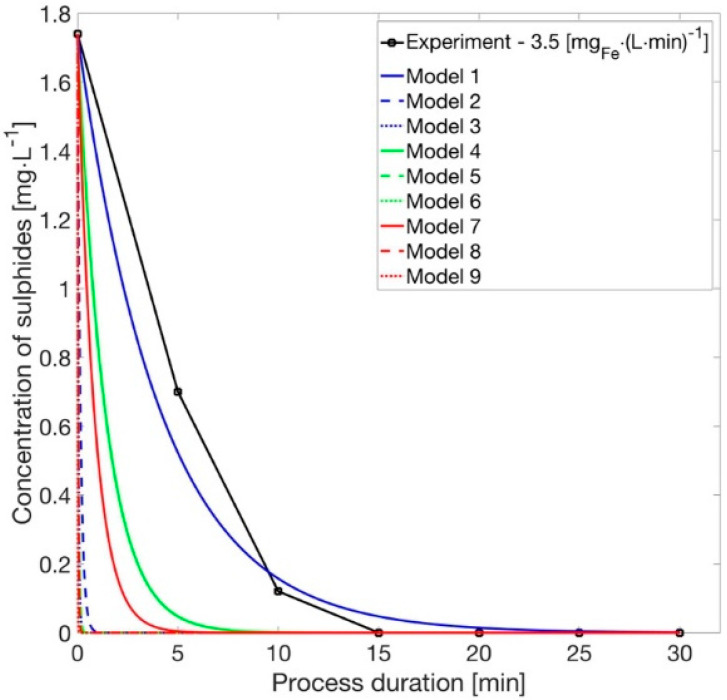
Amount of the sulphides; experimental values and proposed model.

These coefficients in turn correspond to the influence of temperature, feed change, and electrical influence. After implementing Equations (9), (11) and (12) in the numerical code, information on how sulphides are removed from synthetic wastewater (Figure 6) was obtained.

**Figure 7:**
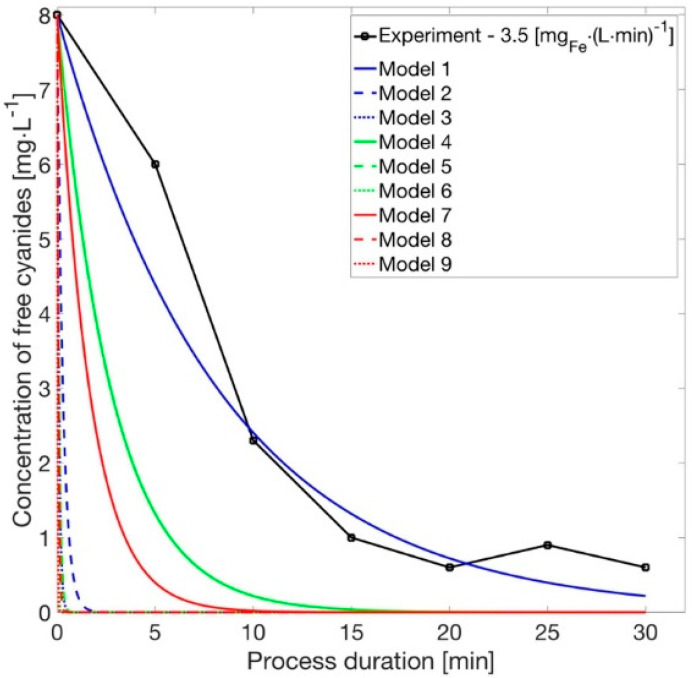
Amount of the free cyanides; experimental values and proposed model.

After bringing about changes in those equations according to the values of free cyanides, we obtain the concentration in a specific period of time (Figure 7).

The information gained was applied to the next steps.

### 3.4. Numerical Simulations and Visual Characteristics of Electrocoagulation Flocks

The computer fluid mechanics were used in this stage for analyzing the process flow in terms of information on the liquid flow in the reactor. A better understanding of the problem related to fluid behavior allows for the proper design of the system and the selection of appropriate materials for its construction. In general, actual flows are three-dimensional, heterogeneous, and anisotropic. There are various methods to simulate these issues, which allow for a statistical description of such diverse flows. These methods should be characterized by adequate accuracy, simplicity, and computational efficiency. Currently, several models are available to simulate turbulent flow in stirred tanks. In the case of computer fluid mechanics, the modeling was provided using Reynolds-averaged Navier–Stokes equations, referred to as the RANS model. In the RANS model, the equations are averaged over a period of time or in an equivalent set of fields. RANS calculations are widely used in engineering practice to predict steady-state solutions. Anisotropy, which is in the nature of flow, introduces key ambiguities into the calculations.

Navier–Stokes equations are used to represent the characteristics of turbulence and to build the basis for describing the flow phenomenon. The chaotic nature of turbulent flows acts as a direct result of non-linear expressions in the N–S equations. These equations are based on the fundamental laws of continuity, conservation of momentum, and energy.

(14) $$\frac{\partial \rho }{\partial t}+\nabla \cdot \left(\rho u\right)=0$$

(15) $$\frac{\partial \rho u}{\partial t}+\nabla \cdot \left(\rho \mathrm{uu}\right)=-\nabla \cdot P$$

(16) $$\frac{\partial \rho e}{\partial t}+\nabla \cdot \left(\mathrm{eu}\right)=-\nabla \cdot \left(u\cdot P\right)-\nabla \cdot q$$

where u, ρ, e, and q are the components of velocity, density, the total energy per unit volume, and heat flux, respectively.

The stress tensor, P for the Newtonian fluid, is defined by:

(17) $$P=p\left(\rho ,T\right)I+\frac{2}{3}\mu \left(\nabla \cdot u\right)I-\mu \left[\left(\nabla u\right)+{\left(\nabla v\right)}^{T}\right]$$

where, p (ρ, T) is the pressure, I is a diagonal unit tensor, T is the temperature, and μ is the dynamic viscosity coefficient. So, the Navier–Stokes equation can be written as:

(18) $$\frac{\partial U_{i}}{\partial t}+U_{j}\frac{\partial U_{i}}{\partial x_{j}}=-\frac{\partial }{\partial x_{i}}\left(\frac{P}{\rho }\right)+\frac{\partial }{\partial x_{j}}\left(v\frac{\partial U_{i}}{\partial x_{j}}\right)$$

The k-ε model is a two-equation method in the RANS approach, where the kinetic energy of the kinetic turbulence and its scattering rate (ε) are used for the transient description. These two parameters are obtained in the flow field by the current solving of modeling equations of their partial modeling.

The standard k-ε model is used for large Reynolds numbers. This model was formulated on the assumption that the Reynolds stress is proportional to the average speed gradient.

The proportional constant is taken as the viscosity of the vortex given as:

(19) $$v_{t}=C_{\mu }\frac{k^{2}}{\varepsilon }$$

where k is the kinetic energy, ε is the rate of dissipation, and C_μ_ is the parameter dependent on the turbulent model k-ε.

The equation showing kinetic turbulence for three-dimensional flows can be presented as follows.

(20) $$k=\frac{1}{2}\left(u^{2}+v^{2}+w^{2}\right)$$

The basic transport equations for the standard k-ε model are given in the following Equations (21) and (22), respectively:

(21) $$\frac{\partial \left(\rho k\right)}{\partial t}+\frac{\partial \left({\rho u}_{i}k\right)}{\partial x_{i}}=\frac{\partial \left({\rho u}_{i}k\right)}{\partial x_{i}}\left(\frac{\mu_{t}}{\sigma_{k}}\cdot \frac{\partial k}{\partial x_{i}}\right)+\rho \cdot \left(P-\varepsilon \right)$$

(22) $$\frac{\partial \left(\rho \varepsilon \right)}{\partial t}+\frac{\partial \left({\rho u}_{i}\varepsilon \right)}{\partial x_{i}}=\frac{\partial \left({\rho u}_{i}k\right)}{\partial x_{i}}\left(\frac{\mu_{t}}{\sigma_{\varepsilon }}\cdot \frac{\partial \varepsilon }{\partial x_{i}}\right)+\rho \cdot \frac{1}{\tau_{d}}\cdot \left(C_{1,\varepsilon }P-C_{2,\varepsilon }\varepsilon \right)$$

where τ_d_ is the time of dissipation rate that characterizes the dynamic process in the energy spectrum and P is the evolution of turbulence, represented as:

(23) $$\tau_{d}=\frac{k}{\varepsilon }$$

(24) $$P=v_{t}\left(\frac{\partial u_{i}}{\partial x_{j}}+\frac{\partial u_{j}}{\partial x_{i}}\right)\frac{\partial u_{j}}{\partial x_{i}}$$

The values of the empirical constants of the standard model k-ε are C_μ_ = 0.09, σ_k_ = 1, σ_ε_ = 1.314, C(1, ε) = 1.44, and C(2, ε) = 1.92.

The standard model k-ε combines reasonable accuracy, computing time economics, and computational stability for a wide range of turbulent flows.

In order to determine the degree of removal of harmful substances from the liquid, coupling between the Fluent and Rocky programs was additionally applied. The latter of these programs has been used to simulate the aggregation of molecules carried by hydroxides onto the liquid surface. This system uses the discrete element method to predict the behavior of molecules during movement in the fluid stream and their interaction. In contrast to the CFD program used, the Rocky program is based on the wireless method. The equations of motion known from the classical mechanics of continuous centers are not solved. Therefore, no additional equations are required, i.e., constitutive equations determining relations σ = σ (ε). The equations of motion for each individual molecule are numerically integral to the time variable, and hence the knowledge of the total force acting on the molecule is required. This force is the result of the interaction of the contact forces and the mass forces.

In the case of the discrete element method all molecules in the Essential Computing System are tracked in terms of Lagrang. Shift equations are computed explicitly

(25) $$m_{p}\frac{dv_{p}}{\mathrm{dt}}=F_{p}^{C}+m_{p}g+F_{p}^{\mathrm{fp}}$$

(26) $$I_{p}\frac{d\omega_{p}}{\mathrm{dt}}=T_{p}$$

These forces that affect molecules can be divided into contact forces (interaction between molecules and molecules and obstacle) and mass forces (gravity or forces associated with the flow of fluid). Generally, to describe these forces, one can use the distribution in two perpendicular directions—the normal direction and the tangent direction.

(27) $$F_{p}^{C}=F_{p,n}^{C}+F_{p,t}^{C}$$

**Figure 8:**
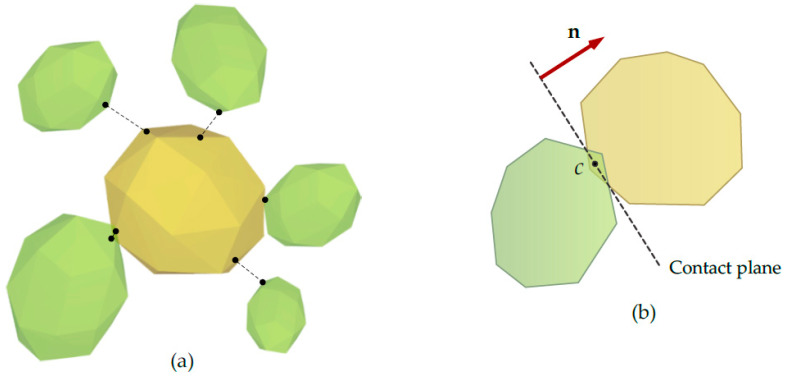
Diagram of the interaction between molecules. (**a**) contact forces (**b**) mass forces.

In the case of the discrete element method code, molecules are usually treated as nondeformable bodies, but it is possible to penetrate them to a certain extent. This is related to the need to determine the normal and tangent direction as a function of the normal permeation of bodies (Figure 8).

**Figure 9:**
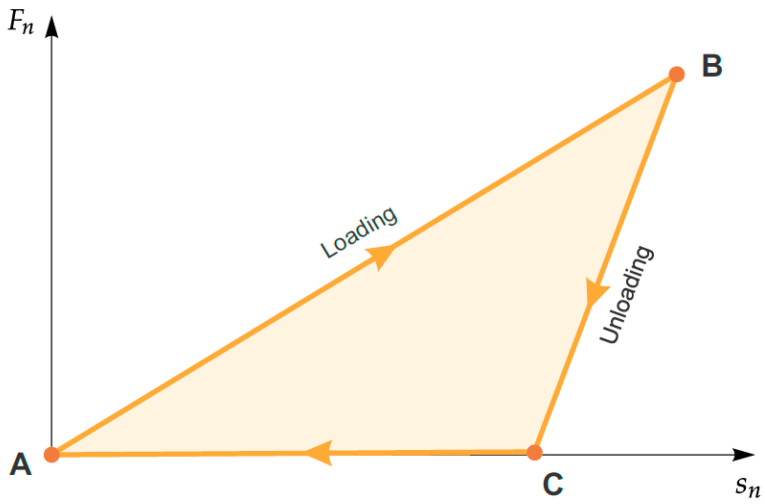
Diagram of the typical hysteresis for the normal force in the case of a linear model.

A typical relief load cycle is illustrated in Figure 9 between points A and B, and there is a loading process (collision) where the body’s normal force of the superposition of bodies increases linearly with the K_nl_ slope. After reaching the maximum overlap, the unloading process follows the B-C line, where the slope is K_nu_. The plastic deformation of the contact between the bodies only exists during the contact phenomenon itself, therefore when this phenomenon does not occur, there are also no residual deformations. The energy that is dissipated during particle collisions in numerical terms can be expressed by the field under the graph of the loading/unloading process.

The normal force that occurs during a collision is calculated from the following equations.

(28) $$F_{p,n}^{C,t}=\min\left(F_{p,n}^{C,t-\mathrm{dt}}+K_{\mathrm{nu}}\cdot {\mathrm{ds}}_{n},K_{\mathrm{nl}}s_{n}\right){\;\mathrm{if}\;\mathrm{ds}}_{n}\geq 0$$

(29) $$F_{p,n}^{C,t}=\max\left(F_{p,n}^{C,t-\mathrm{dt}}+K_{\mathrm{nu}}\cdot {\mathrm{ds}}_{n},0.0001K_{\mathrm{nl}}s_{n}\right){\;\mathrm{if}\;\mathrm{ds}}_{n}<0$$

(30) $${\mathrm{ds}}_{n}=s_{n}^{t}-s_{n}^{t-\mathrm{dt}}$$

**Figure 10:**
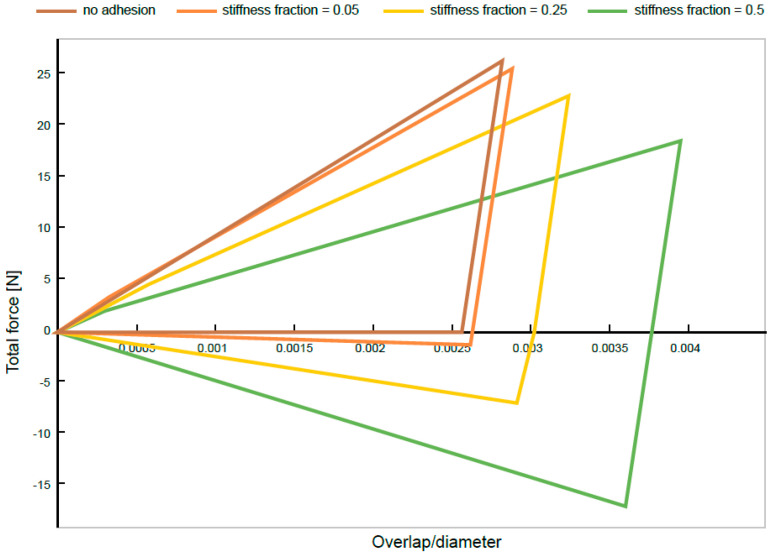
An example of a force diagram during particle collisions for different adhesion stiffness coefficients.

This model describes non-attracting materials, however during the process, most of the molecules are conglomerated, hence the use of a modified model additionally describing the phenomenon of adhesion is required (Figure 10). The load/unload plot for a model shows the adhesion between molecules.

The linear adhesion model shows behavior that reminds one of a spring with a linear characteristic with the ability to attract. The adhesion force is zero if the distance between the bodies is greater than the minimum distance defined for this force. It grows in proportion to the difference between this distance and the current depth of overlap. The proportionality factor is referred to as the stiffness of the adhesion force.

(31) $$F_{n,\mathrm{adh}}=\{\begin{array}{c} 0\;\;\;\;\;\;\;\mathrm{if}\;\;\;\;\;\;\;\;\;-s_{n}\geq \delta_{\mathrm{adh}}\\ r_{\mathrm{adh}}\cdot K_{\mathrm{nl}}\left(s_{n}+\delta_{\mathrm{adh}}\right),\;\mathrm{for}\;\mathrm{other}\;\mathrm{cases} \end{array}$$

When CFD and DEM analysis are combined, the effect of fluid motion on the kinematics of the molecule is achieved by the expression F_p_^fp^ in the equation describing linear motion. The force associated with the pressure gradient is expressed by the formula:

(32) $$F_{\nabla p}=-V_{p}\nabla p$$

The lifting force can be saved as:

(33) $$F_{D}=\beta \left(u-v\right)$$

This force differs for each molecule; therefore, the coefficient β depends on the volume fraction of the cell in which the force is calculated.

(34) $$\beta =\frac{3}{4}\frac{\left(1-\alpha_{f}\right)\rho_{f}C_{D}}{d_{p}}\left|v-u\right|$$

### 3.5. Computational Fluid Dynamics Simulations

**Figure 11:**
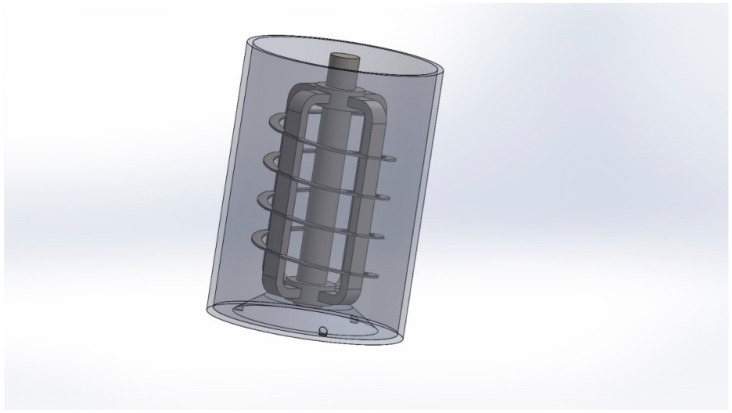
A simplified reactor geometry model.

The simulation included a suitably simplified reactor model, in which ARMCO steel electrodes act as partitions in an open tank. The reactor geometry, fluid volume, and electrode baffles were modeled as separate regions in Autodesk Inventor software (Figure 11).

Then these geometries were imported into the FLUENT environment for pre-processing. The extensive interfaces between the contacting fluid areas and boundary conditions were created directly in the geometric module of the ANSYS program. The grid was generated to discretize the computational domain into small control volumes, where the basic equations of fluid mechanics were approximated by means of computer numerical calculations. The grid of the system under consideration contained two main zones: The fluid area in the tank and the agitator area.

**Figure 12:**
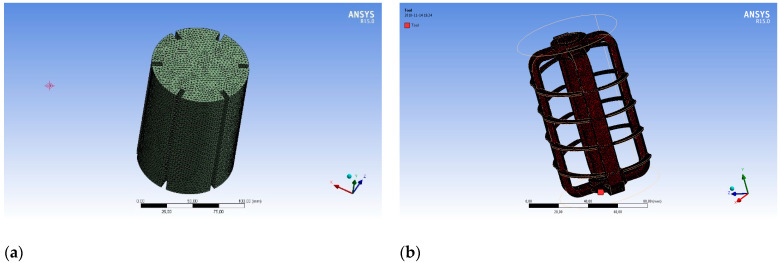
Grid generated consisting of elements selected for CFD analysis: (**a**) Generated mesh of finite elements; (**b**) surface elements modeling the cathode within a computational domain.

They have been modeled as separate domains that interact with each other. A grid of small-sized elements was used to increase the stability and accuracy of calculations. Figure 12a,b shows the generated grid consisting of elements selected for the CFD analysis.

The simulations were carried out using the solver using a steady state based on pressure and absolute velocity conditions with the force of gravity in the negative direction of the *Y* axis. The created domain was then configured as a viscous type in the standard model k-ε with standard boundary functions. The reservoir fluid was chosen for water density of ρ = 998.2 kg·m^−3^ and a constant dynamic viscosity of μ = 0.001 kg/m·s. Cell zone conditions included an interface between the agitator and the fluid that consisted of the agitator surface and the fluid area around the agitator. The mesh surfaces and contact areas were confirmed to be the exact points at which interactions occurred. The movement of the rotor zone in the fluid area was modeled using the Arbitrary Mesh Interface option, which combines both stationary and mobile mesh calculations. In this case, both zones must consist of well-defined borders. The motion zone consists of a mixer and a shaft that rotate at 20 rpm along the *Y* axis. The fluid zone in the tank with the baffles and walls of the tank is set as stationary. The simulation was configured before commissioning using a hybrid technique. The other parameters used in the simulation are shown in Table 2.

**Figure 13:**
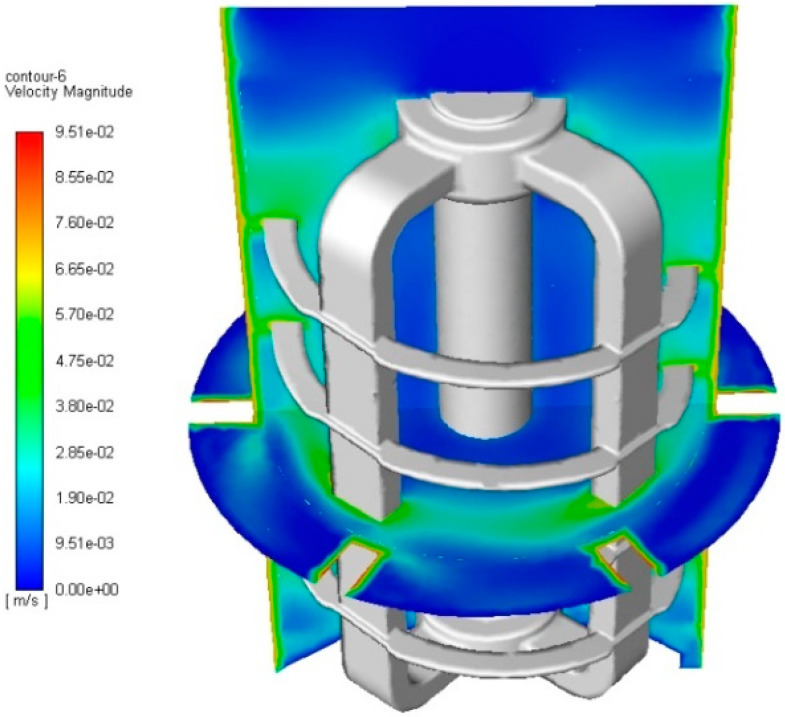
Fluid velocity field within the agitator.

On the basis of numerical simulation, a post-processing analysis was performed, taking into account the field of fluid velocity, power lines, and the distribution of the field of current intensity. Figure 13 shows that in the entire working area of the mixer, medium velocity values of the fluid are present. Only in the locations of divisions in the form of electrodes and cathode shaft were areas with a low-velocity gradient created. This may be due to the relatively low agitator speed.

**Figure 14:**
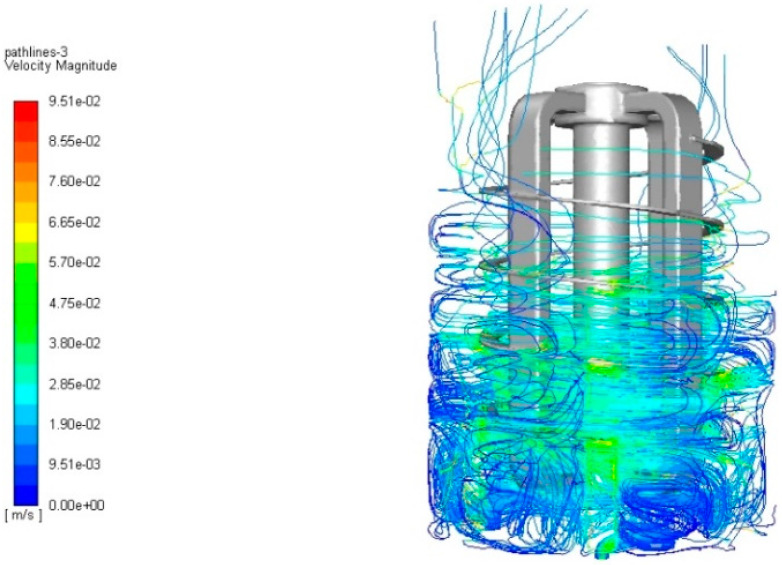
Liquid velocity jets showing the turbulent flow generated by the rotary motion of the stirrer.

**Figure 15:**
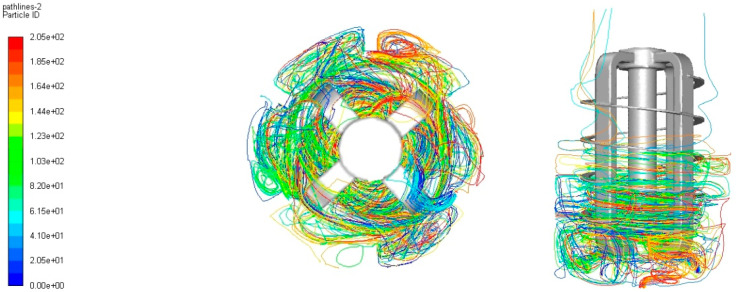
Trajectory of particle movement inside the reactor—view in a plane perpendicular and parallel to the axis of the cathode.

On observing the fluid flow lines within the agitator’s work (Figure 14 and Figure 15) it can be seen that they are characterized by considerable turbulence, which is the result of using a stirrer in the reactor, which is also a cathode in electrocoagulation.

**Figure 16:**
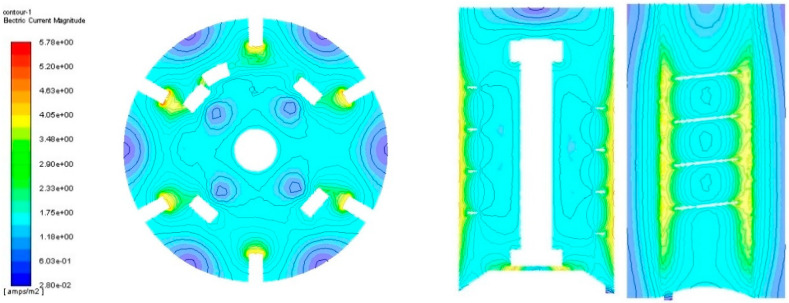
Area of current distribution in various planes to the axis of the electrode.

The post-processing analysis taking into account a fluid velocity field, streamline, and velocity vectors was performed based on numerical simulation. Regarding the fluid flows within the operated mixer (Figure 16) it was observed that they were not characterized by significant turbulence.

However, this type of agitator is not intended to force the flow, but only prevents it from disappearing. It is expected that the generated flow will cause the solution molecules to increase towards the outflow, while the sulphides and cyanides will be removed from the liquid during the flow. A higher concentration of the solution in the lower parts of the tank compared to the upper areas caused by the lack of influence of the mixer in this area may be problematic due to the deposition of waste on the bottom of the device. These observations should be confirmed experimentally, and any confirmation of this phenomenon will require some structural modifications.

These numerical simulations on the electrocoagulation and electroflotation processes were designed to be applied to coke oven wastewater treatment. The virtual model of a stirred tank was developed. The simulations were performed with a solver using a steady state based on pressure and absolute velocity with the use of gravity towards the negative values of the *Y* axis. The post-processing analysis taking into account a fluid velocity field, a streamline, and velocity vectors was conducted based on numerical simulation. By observing fluid flows within the operated mixer it was noticed that they were not characterized by significant turbulence. That was likely a result of using a low rotational speed (5 rpm). However, this type of mixer was not used to force a flow but to prevent its disappearance. It was expected that the generated flow would cause lifting of the solution’s particles towards the outflow, and during a flow it would eliminate sulphides and cyanides in the fluid. These observations need to be confirmed experimentally, and potential confirmation of this process would require some design modifications. 

The economic analysis presented for the replacement of conventional coagulation with electrocoagulation for coke oven wastewater showed that if the process was applied to plants with an external power supply, its economics were not yet justified, but if a coke oven plant possessed its own power supply, the electrocoagulation treatment was more beneficial than conventional coagulation. However, it is worth noting, that the process of electrocoagulation is also used in other industries [29,30,31,32,33,34,35,36,37]. The process has considerable potential for practical applications.

## 4. Conclusions

The characterization of electrocoagulation flocks was performed as the first part of the research work. The most important elements of the characterization were measurements of the particle size distribution and the characteristic of a dedicated device for sedimentation/flotation. Then, an estimated concept of the geometry and fluid flow of the continuous-flow electrocoagulation reactor was presented. Eventually, the mathematical model and numerical simulations of the electrocoagulation and electroflotation processes were presented. Numerical simulations and visual characteristics of electrocoagulation flocks were examined, and on the basis of these results, the virtual stirred tank electrocoagulation reactor was designed and numerically tested, including an economic assessment. Based on the results achieved, the following conclusions may be formulated:

- Mathematical modelling fed with experimental results obtained for small-scale electrocoagulation reactors is a powerful tool suitable to optimize the process;
- The developed models may be successfully adapted to design an industrial-scale electrocoagulator, and, together with the scaling-up procedure, they should allow for determination of its operational parameters and desired geometry;
- Electrocoagulation has huge potential to become a commonly used technique for water/wastewater treatment;
- The main barriers currently identified for the technology are related to the high costs of energy and relatively low environmental charges, which affect the economy of the process.

In the case of such a project of designing systems for treating wastewater from coke ovens, mathematical modelling is a crucial part of the project that allows for cheaper and more effective design of complex processes. The conducted research shows that the mathematical modelling fed with experimental results obtained for small-scale electrocoagulation reactors is a powerful tool suitable to optimize the process. It brings benefits and allows for the developed models to be easily scaled-up, even to the industrial scale.

## 5. Patents

The reactor scheme is classified due to a patent procedure in the Polish Patent Office.

## Figures and Tables

**Table 1:** Electrical parameters for modelling.

Model Number	U [V]	I [A]
1	2	0.15
2		0.80
3		1.45
4	6	0.15
5		0.80
6		1.45
7	10	0.15
8		0.80
9		1.45

**Table 2:** Parameters of the electrocoagulation process in numerical simulation.

Parameter	Value
Process time	30 min
The volume to be replaced	1 L
Rotation of the electrode	20 rpm
Voltage	2 V
Current	1.45 A
